# Supplementary material for: Effects of short‐term unloading and active recovery on human motor unit properties, neuromuscular junction transmission and transcriptomic profile
Source: J Physiol. 2022 Sep 27;600(21):4731–51. doi: 10.1113/JP283381 (PMC9828768; doi:10.1113/JP283381)
Supplement: Supplementary file 1 — Statistical Summary Document [file TJP-600-4731-s003.docx]

**Manuscript Title:** Effects of short-term unloading and active recovery on human motor unit properties, neuromuscular junction transmission and transcriptomic profile

**Authors:** Fabio Sarto, Daniel W. Stashuk, Martino V. Franchi, Elena Monti, Sandra Zampieri, Giacomo Valli, Giuseppe Sirago, Julián Candia, Lisa M. Hartnell, Matteo Paganini, Jamie S. McPhee, Giuseppe De Vito, Luigi Ferrucci, Carlo Reggiani and Marco V. Narici

**Underlying hypothesis:** This investigation tests the hypothesis that 10-day unilateral lower limb suspension (ULLS), would be sufficient to (i) cause neuromuscular junction (NMJ) molecular alterations, possibly resulting in NMJ transmission stability impairment and (ii) increase motor unit MUP complexity and decrease MUs firing rate, in association with loss of muscle function and (iii) 21-day active recovery based on resistance exercise would counteract these neuromuscular changes.

**Definitions of ‘n’:**

For all Questions: n = 11

|  |  |  |  |  |  |  |  |  |  |  |  |  |
| --- | --- | --- | --- | --- | --- | --- | --- | --- | --- | --- | --- | --- |
| **Experimental question number** | **Finding/ conclusion** | **Experimental variable** | **Mean value** | **SD** | **n** | **P** | **Units** | **Data comparisons** | **Statistical test** | **Any other variable** | **Figure/ table in which data are presented** | **Comment** |
| 1. Impact of ULLS and AR on in vivo muscle morphology and function | ULLS resulted in a reduction in muscle size and function. AR restored to baseline values | Quadriceps CSA | LS0: 57.33  LS10: 54.70  AR21: 64.83 | LS0: 6.39  LS10: 6.17  AR21: 7.58 | 11 | LS0vsLS10: 0.0074  LS10vsAR21: **<0.0001**  LS0vsAR21: **<0.0001** | cm^2^ | LS0 vs LS10 vs AR21 | Repeated-measures one-way ANOVA |  | 1 |  |
|  |  | Vastus lateralis CSA | LS0: 18.47  LS10: 17.69  AR21: 21.80 | LS0: 2.77  LS10: 2.53  AR21: 3.23 | 11 | LS0vsLS10: 0.0384  LS10vsAR21: **<0.0001**  LS0vsAR21: **<0.0001** | cm^2^ | LS0 vs LS10 vs AR21 | Repeated-measures one-way ANOVA |  | 1 |  |
|  |  | MVC | LS0: 797.0  LS10: 563.2  AR10: 678.8  AR21: 791.7 | LS0: 110.9  LS10: 101.2  AR10: 86.87  AR21: 106.0 | 11 | LS0vsLS10: **<0.0001**  LS10vsAR10: **<0.0001**  LS10vsAR21: 0.0003  AR10vsAR21: **<0.0001**  LS0vsAR10: 0.0038  LS0vsAR21: 0.9951 | N | LS0 vs LS10 vs AR10 vs AR21 | Repeated-measures one-way ANOVA |  | 1 |  |
|  |  | TTP63% | LS0: 0.158  LS10: 0.244  AR21: 0.139 | LS0: 0.034  LS10: 0.083  AR21: 0.042 | 11 | LS0 vs LS10: 0.0043  LS10vsAR21: 0.0030  LS0vsAR21: 0.1365 | s | LS0 vs LS10 vs AR21 | Repeated-measures one-way ANOVA |  | 1 |  |
|  |  | Activation capacity | LS0: 93.20  LS10: 87.59  AR21: 92.61 | LS0: 3.09  LS10: 5.25  AR21: 4.36 | 11 | LS0 vs LS10: 0.0195  LS10vsAR21: 0.0004  LS0vsAR21: 0.9009 | % | LS0 vs LS10 vs AR21 | Repeated-measures one-way ANOVA |  | 1 |  |
|  |  | Specific force | LS0: 13.95  LS10: 10.32  AR21: 12.24 | LS0: 1.714  LS10: 1.696  AR21: 1.225 | 11 | LS0 vs LS10: **<0.0001**  LS10vsAR21: 0.0159  LS0vsAR21: 0.0327 | N/ cm^2^ | LS0 vs LS10 vs AR21 | Repeated-measures one-way ANOVA |  | 1 |  |
| 2. Impact of ULLS and AR on NMJ molecular and transmission stability? | Unloading cause NMJ molecular alteration, but without NMJ transmission stability impairment. AR reversed NMJ molecular alterations | CAF | LS0: 4251.1  LS10: 4494.5  AR21: 4569.6 | LS0: 764.3  LS10: 908.4  AR21: 998.6 | 11 | LS0 vs LS10: 0.0380  LS10vsAR21: 0.9109  LS0vsAR21: 0.2547 | pg/ml | LS0 vs LS10 vs AR21 | Repeated-measures one-way ANOVA |  | 8 |  |
|  |  | NMJ genes | See Data S1 and S3 | | | | | LS0 vs LS10 vs AR21 | PCA and GSEA |  | 4 and 5 | n=9 at AR21; but statistics employed handle missing values |
|  |  | NF Jiggle  10% MVC | LS0: 9.47  LS10: 9.63  AR21: 8.3 | LS0: 0.55*  LS10: 0.56*  AR21: 0.51* | 11 | LS0 vs LS10: 0.7138  LS10vsAR21: 0.0012  LS0vsAR21: 0.0016 | % | LS0 vs LS10 vs AR21 | Generalized linear mixed model |  | 3 |  |
|  |  | NF Jiggle  25% MVC | LS0: 13.5  LS10: 13.7  AR21: 13.6 | LS0: 1.04*  LS10: 1.04*  AR21: 1.03* | 11 | LS0 vs LS10: 1.000  LS10vsAR21: 1.000  LS0vsAR21: 1.000 | % | LS0 vs LS10 vs AR21 | Generalized linear mixed model |  | 3 | n=10 at LS10; but statistics employed handles missing values |
|  |  | NF Jitter 10% MVC | LS0: 27.9  LS10: 26.5  AR21: 28.2 | LS0: 1.6*  LS10: 1.5*  AR21: 1.6* | 11 | LS0 vs LS10: 0.3297  LS10vsAR21: 0.7176  LS0vsAR21: 0.1665 | µs | LS0 vs LS10 vs AR21 | Generalized linear mixed model |  | 3 |  |
|  |  | NF Jitter 25% MVC | LS0: 35.7  LS10: 34.9  AR21: 36.4 | LS0: 2.1*  LS10: 2.0*  AR21: 2.1* | 11 | LS0 vs LS10: 1.000  LS10vsAR21: 0.5442  LS0vsAR21: 1.000 | µs | LS0 vs LS10 vs AR21 | Generalized linear mixed model |  | 3 | n=10 at LS10; but statistics employed handles missing values |
| 3. Impact of LLS and AR on MUP and near fibre MUP properties | We observed changes in MUP complexity and firing rate with unloading that are mostly restored with AR | MUP Area 10% MVC | LS0: 882  LS10: 748  AR21: 634 | LS0: 44*  LS10: 46*  AR21: 42* | 11 | LS0 vs LS10: 0.093  LS10vsAR21: 0.0084  LS0vsAR21: **<0.0001** | µV · ms | LS0 vs LS10 vs AR21 | Generalized linear mixed model |  | 2 |  |
|  |  | MUP Area 25% MVC | LS0: 1008  LS10: 952  AR21: 887 | LS0: 45*  LS10: 44*  AR21: 44* | 11 | LS0 vs LS10: 0.1044  LS10vsAR21: 0.1044  LS0vsAR21: 0.0001 | µV · ms | LS0 vs LS10 vs AR21 | Generalized linear mixed model |  | 2 | n=10 at LS10; but statistics employed handles missing values |
|  |  | MUP Duration  10% MVC | LS0: 10.47  LS10: 10.30  AR21: 8.79 | LS0: 0.42*  LS10: 0.42*  AR21: 0.3* | 11 | LS0 vs LS10: 0.6391  LS10vsAR21: **<0.0001**  LS0vsAR21: **<0.0001** | ms | LS0 vs LS10 vs AR21 | Generalized linear mixed model |  | 2 |  |
|  |  | MUP Duration 25% MVC | LS0: 10.18  LS10: 10.74  AR21: 8.85 | LS0: 0.44*  LS10: 0.48*  AR21: 0.34* | 11 | LS0 vs LS10: 0.1429  LS10vsAR21: **<0.0001**  LS0vsAR21: **<0.0001** | ms | LS0 vs LS10 vs AR21 | Generalized linear mixed model |  | 2 | n=10 at LS10; but statistics employed handles missing values |
|  |  | MUP turns  10% MVC | LS0: 3.34  LS10: 3.46  AR21: 3.23 | LS0: 0.13*  LS10: 0.14*  AR21: 0.11* | 11 | LS0 vs LS10: 0.6465  LS10vsAR21: 0.1453  LS0vsAR21: 0.6465 | n | LS0 vs LS10 vs AR21 | Generalized linear mixed model |  | 2 |  |
|  |  | MUP turns 25% MVC | LS0: 2.99  LS10: 3.53  AR21: 3.12 | LS0: 0.09*  LS10: 0.14*  AR21: 0.10* | 11 | LS0 vs LS10: **<0.0001**  LS10vsAR21: **<0.0001**  LS0vsAR21: 0.1627 | n | LS0 vs LS10 vs AR21 | Generalized linear mixed model |  | 2 | n=10 at LS10; but statistics employed handles missing values |
|  |  | IDImean 10% MVC | LS0: 134  LS10: 148  AR21: 126 | LS0: 5*  LS10: 5*  AR21: 5* | 11 | LS0vsLS10: 0.0001  LS10vsAR21: **<0.0001**  LS0vsAR21: 0.0075 | ms | LS0 vs LS10 vs AR21 | Generalized linear mixed model |  | 2 |  |
|  |  | IDImean 25% MVC | LS0: 129  LS10: 138  AR21: 134 | LS0: 6*  LS10: 6*  AR21: 6* | 11 | LS0vsLS10: 0.0304  LS10vsAR21: 0.2467  LS0vsAR21: 0.228 | ms | LS0 vs LS10 vs AR21 | Generalized linear mixed model |  | 2 | n=10 at LS10; but statistics employed handles missing values |
|  |  | NF Duration 10% MVC | LS0: 2.05  LS10: 2.22  AR21: 2.62 | LS0: 0.13*  LS10: 0.15*  AR21: 0.19* | 11 | LS0vsLS10: 0.0604  LS10vsAR21: **<0.0001**  LS0vsAR21: **<0.0001** | ms | LS0 vs LS10 vs AR21 | Generalized linear mixed model |  | 3 |  |
|  |  | NF Duration 25% MVC | LS0: 2.06  LS10: 2.25  AR21: 2.42 | LS0: 0.11*  LS10: 0.13*  AR21: 0.15* | 11 | LS0vsLS10: 0.0316  LS10vsAR21: 0.0316  LS0vsAR21: **<0.0001** | ms | LS0 vs LS10 vs AR21 | Generalized linear mixed model |  | 3 | n=10 at LS10; but statistics employed handles missing values |
|  |  | NF count 10% MVC | LS0: 1.1  LS10: 1.23  AR21: 1.19 | LS0: 0.04*  LS10: 0.05*  AR21: 0.05* | 11 | LS0vsLS10: 0.0066  LS10vsAR21: 0.4222  LS0vsAR21: 0.0144 | n | LS0 vs LS10 vs AR21 | Generalized linear mixed model |  | 3 |  |
|  |  | NF count 25% MVC | LS0: 1.14  LS10: 1.27  AR21: 1.26 | LS0: 0.04*  LS10: 0.05*  AR21: 0.05* | 11 | LS0vsLS10: 0.0014  LS10vsAR21: 0.8742  LS0vsAR21: 0.0014 | n | LS0 vs LS10 vs AR21 | Generalized linear mixed model |  | 3 | n=10 at LS0; but statistics employed handles missing values |
| 4. What are the underlying mechanisms for MUP properties changes? | Initial axonal damage, partial denervation and ion channels remodelling are possible mechanisms | Slow fibre type percentage | LS0: 33.81  LS10: 31.12  AR21: 49.10 | LS0: 11.73  LS10: 13.12  AR21: 16.85 | 11 | LS0vsLS10: 0.6078  LS10vsAR21: 0.0009  LS0vsAR21: 0.0035 | % | LS0 vs LS10 vs AR21 | Mixed-effects repeated-measures one-way ANOVA |  | 7 | n=9 at AR21; but statistics employed handles missing values |
|  |  | Fast fibre type percentage | LS0: 66.19  LS10: 68.88  AR21: 66.19 | LS0: 11.73  LS10: 13.12  AR21: 16.85 | 11 | LS0vsLS10: 0.6078  LS10vsAR21: 0.0009  LS0vsAR21: 0.0035 | % | LS0 vs LS10 vs AR21 | Mixed-effects repeated-measures one-way ANOVA |  | 7 | n=9 at AR21; but statistics employed handles missing values |
|  |  | Slow fibre type variability | LS0: 0.192  LS10: 0.203  AR21: 0.203 | LS0: 0.024  LS10: 0.042  AR21: 0.035 | 11 | LS0vsLS10: 0.7660  LS10vsAR21: 0.9999  LS0vsAR21: 0.6126 | % | LS0 vs LS10 vs AR21 | Mixed-effects repeated-measures one-way ANOVA |  | 7 | n=9 at AR21; but statistics employed handles missing values |
|  |  | Fast fibre type variability | LS0: 0.212  LS10: 0.222  AR21: 0.204 | LS0: 0.0352  LS10: 0.036  AR21: 0.031 | 11 | LS0vsLS10: 0.6434  LS10vsAR21: 0.3901  LS0vsAR21: 0.8818 | % | LS0 vs LS10 vs AR21 | Mixed-effects repeated-measures one-way ANOVA |  | 7 | n=9 at AR21; but statistics employed handles missing values |
|  |  | Slow fibre grouping | LS0: 0.988  LS10: 0.408  AR21: 7.744 | LS0: 2.795  LS10: 1.153  AR21: 15.52 | 9 | LS0vsLS10: >0.9999  LS10vsAR21: 0.6339  LS0vsAR21: 0.9519 | % | LS0 vs LS10 vs AR21 | Friedman test |  | 7 | n=9 at AR21; but statistics employed handles missing values |
|  |  | Fast fibre grouping | LS0: 14.69  LS10: 17.07  AR21: 3.278 | LS0: 13.71  LS10: 12.54  AR21: 3.989 | 11 | LS0vsLS10: 0.8406  LS10vsAR21: 0.0209  LS0vsAR21: 0.0336 | % | LS0 vs LS10 vs AR21 | Mixed-effects repeated-measures one-way ANOVA |  | 7 | n=9 at AR21; but statistics employed handles missing values |
|  |  | NCAM positive fibres | LS0: 0.045  LS10: 0.104 | LS0: 1.155  LS10: 1.596 | 11 | 0.031 | % | LS0 vs LS10 | Wilcoxon test |  | 8 |  |
|  |  | Ion channels genes | See Data S2 and S3 | | | | | LS0 vs LS10 vs AR21 | PCA and GSEA |  | 6 | n=9 at AR21; but statistics employed handles missing values |
|  |  | Neurofilament light chain | LS0: 5.518  LS10: 8.073  AR21: 4.749 | LS0: 1.801  LS10: 2.855  AR21: 1.180 | 11 | LS0vsLS10: 0.0013  LS10vsAR21: 0.0012  LS0vsAR21: 0.0725 | ng/L | LS0 vs LS10 vs AR21 | Repeated-measures one-way ANOVA |  | 8 |  |

*Data presented as SEM instead of SD. Indeed, Generalized Linear Mixed Models (used to perform statistical analysis on iEMG) does not calculate SDs, as the underlying assumption is that not all subjects contribute to the overall variance for a single variable.
